# BRAVA: A randomized controlled trial of a brief group intervention for youth with suicidal ideation and their caregivers

**DOI:** 10.1186/s13034-025-00941-1

**Published:** 2025-07-16

**Authors:** Allison Kennedy, Clare Gray, Nicole Sheridan, Leigh Dunn, Jayme Stewart, Stéphanie Drouin, Hannah Elliott, Ademola Adeponle, Nicholas Barrowman, Ewa Sucha, Mario Cappelli, Mark L. Norris, Mona Jabbour, Paula Cloutier

**Affiliations:** 1 Children’s Hospital of Eastern Ontario (CHEO), 401 Smyth Road, Ottawa, ON K1H 8L1 Canada; 2 CHEO Research Institute, 401 Smyth Road, Ottawa, ON K1H 8L1 Canada; 3 University of Ottawa, 451 Smyth Road, Ottawa, ON K1H 8L1 Canada; 4 Memorial University of Newfoundland, PO Box 4200, St. John’s, NL A1C 5S7 Canada; 5 University of British Columbia, 3333 University Way, Kelowna, BC V1V 1V7 Canada; 6 Carleton University, 1125 Colonel By Drive, Ottawa, ON K1S 5B6 Canada; 7 Knowledge Institute for Child and Youth Mental Health and Addictions, 695 Industrial Avenue, Ottawa, ON K1G 0Z1 Canada

**Keywords:** Suicidal ideation, Treatment, Adolescents, Caregivers, Effectiveness study, Randomized controlled trial

## Abstract

**Objectives:**

Suicide is the second leading cause of mortality among Canadian youth. As wait times for mental health (MH) support have increased, adolescents with mild-to-moderate suicidal ideation (SI) are waiting longer for support compared to those with more acute SI. Building Resilience and Attachment in Vulnerable Adolescents (BRAVA) is a 6-week virtual group intervention designed to provide support to adolescents with mild to moderate SI and their caregivers. We conducted a randomized controlled trial to assess the efficacy of BRAVA in reducing symptoms of SI, depression, and anxiety in adolescents, and improving life stress in caregivers.

**Design/Methods:**

Outcome measures were administered to both groups [BRAVA, Enhanced Treatment-as-Usual (ETU)] at intake and exit, and at 3-month follow-up (BRAVA only) for SI (primary outcome), anxiety and depression (adolescent), perceived stress (youth and caregiver), attachment and family functioning (caregiver). SI was measured using Suicidal Ideation Questionnaire Junior. Intention to treat (ITT) analysis was performed for youth and caregiver cohorts.

**Results:**

Ninety-nine eligible youth presenting with mild-to-moderate SI and their caregivers were recruited from hospital and community MH services. Families were randomized to BRAVA (*n* = 50) or ETU (*n* = 49). Adolescents were on average 14.6 years old, mostly female (64%), and of European racial heritage (44%). In ITT analysis, both BRAVA and ETU groups improved in youth SI from intake to exit, with no statistically significant differences between groups at exit. Sensitivity analysis without multiple imputations demonstrated a significant difference in SI scores at exit between the groups, where improvements in the BRAVA group were maintained at 3-month follow-up. Significant differences between groups on youth perceived stress, and depression and anxiety scores were also observed in BRAVA participants at post-treatment compared to the ETU control group. No statistically significant differences were observed for any caregiver outcomes measured except a trend for improved perceived caregiver stress in the BRAVA group post-treatment.

**Conclusions:**

BRAVA was associated with significantly greater improvements in anxiety/depression and adolescent perceived stress compared to ETU. Although the intervention did not result in significant caregiver reported improvements, group cohesion and treatment satisfaction were high for both youth and caregivers.

**Clinical trial registration:**

BRAVA: Building Resilience and Attachment in Vulnerable Adolescents (BRAVA); https://clinicaltrials.gov/: NCT04751968.

**Supplementary Information:**

The online version contains supplementary material available at 10.1186/s13034-025-00941-1.

## Background

Suicide is the second leading cause of death for adolescents in Canada and the third leading cause in the United States, with rates having increased in recent years [1, 2]. Suicide rates for American adolescents increased significantly from 1999 to 2020 across all methods [3]. During adolescence, many individuals experience suicidal ideation (SI) with roughly one-third of adolescents who experience SI developing a suicide plan, of whom 60% escalate to a suicide attempt (SA) within the first year of SI onset [4, 5]. The 2023 Youth Risk Behavior Survey conducted by the US Center for Disease Control found that, in the last 12 months, 27.1% of female students and 14.1% of male students seriously considered suicide; 12.6% of females and 6.4% of males attempted suicide [6]. Given the high mortality and morbidity of adolescent suicidal behavior, numerous studies have evaluated interventions targeting this behaviour. These interventions have varied in modality, intensity and theoretical orientations with modest outcomes at best.

Itzhaky and colleagues included 30 studies reporting randomized control trials (RCTs) over 26 years in a systematic review and meta-analysis of interventions to reduced suicide risk [7]. Collectively, these interventions showed little effectiveness relative to control treatments. A wide range of interventions using various models demonstrated modest superiority over control interventions, including Dialectical Behavior Therapy (DBT) and some CBT and family-based approaches.

DBT has accumulated the most evidence in reducing SI and SA in adolescents [8, 9]. DBT is a comprehensive intervention involving manualized adolescent and caregiver groups, individual therapy, and crisis support, typically lasting 16 to 24 weeks. A systematic review and meta-analysis regarding the efficacy of DBT found large pre-post treatment effects and small-to-moderate effects relative to control groups in reducing self-harm and SI in 12- to 19-year-olds [8]. For example, Mehlum and colleagues compared DBT-A (DBT for Self-Harming Adolescents) to Enhanced Treatment as Usual (ETU; at least weekly non-standardized outpatient treatment sessions) in a sample of adolescents with recent and repetitive suicidal and self-harming behaviour [10]. The DBT-A treatment group had a more rapid recovery in SI and depressive symptoms and a stronger long-term reduction in self-harm.

Family-based treatments have also demonstrated efficacy in reducing adolescent suicidal behavior, with mixed results [11–15]. A transtheoretical systematic review of studies examining the efficacy of family therapy in adolescents with depressive disorder found that family therapy was more effective in reducing SI but not depression relative to treatment-as-usual or other comparison psychotherapies (e.g., CBT) [15]. Attachment Based Family Therapy (ABFT), which aims to repair parent-child relationship ruptures and increase the security of attachment, is another promising family-based intervention [12, 16]. Primarily a process-oriented, emotion-focused treatment, guided by a semi-structured treatment protocol, it incorporates individual and family sessions, weekly monitoring, and 24/7 crisis response. Three months of ABFT significantly reduced adolescent SI relative to ETU (facilitated referral to outpatient treatment bolstered by safety monitoring) in a sample of adolescents with clinically significant SI and at least moderate depressive symptoms [12]. In a subsequent RCT study, ABFT did not outperform Family-Enhanced Nondirective Supportive Therapy which focused primarily on individual sessions with the adolescent in reducing SI and the authors concluded that ABFT could be fortified by integrating cognitive and emotional skills such as parent or adolescent psychoeducation, CBT, or DBT techniques [13].

Asarnow and colleagues developed the SAFETY Program, a multi-faceted adolescent treatment program aimed at reducing risk of SA which emphasizes strengthening protective supports within the family and other social systems and building skills in youths and parents that lead to safer behaviors and stress reactions [11]. Drawing on social-ecological, cognitive-behavioral and DBT-informed family interventions, it incorporates safety planning and individual, parent, and family sessions, including some in-home sessions. The SAFETY Program significantly reduced SA risk at the 3-month follow-up point compared to ETU for adolescents with recent SA or non-suicidal self-injury as a primary problem along with repetitive self-harm.

The aforementioned interventions are resource-intensive, typically requiring individual intervention or lengthy duration (e.g., 16 weeks for DBT). Demand for adolescent MH services is high in the US and Canada [17–20] and, while an essential component in a system of care, resource-intensive treatments run the risk of lengthy wait-times. Interventions must be delivered promptly to minimize adolescent, family, and system burden of suicidality. Clark et al. (2018) analyzed data from the English Improving Access to Psychological Therapies program to explore organizational factors impacting service outcomes [21]. They found that services with shorter waiting times (i.e., four to six weeks) between initial assessment and treatment initiation were generally associated with better patient engagement and treatment effectiveness for depression and anxiety disorders [21].

Brief interventions, defined as 6–12 sessions [22]for adolescent suicidality are essential to meet burgeoning service demands. Promising brief interventions for SI have emerged in recent years. Adini-Spigelman et al. (2024) developed a brief suicide crisis intervention based on Interpersonal Psychotherapy for Adolescents (IPT-A-SCI) for children and adolescents with a history of SI or SA [23]. Five weeks of individual therapy with parental involvement followed by four personalized emails resulted in significant decreases in SI, suicide behaviors, and depression and anxiety symptoms post-intervention in a clinical non-inferiority trial. IPT-A-SCI and treatment-as-usual (integrative psychotherapy lasting 12 to 30 weeks) resulted in comparable reductions in SI, demonstrating that a brief intervention can be as efficacious as a lengthier one. The authors interpreted comparable improvement in the control group as potentially related to regular contact with research assistants (RA) and the hope of future treatment. To their point, Itzhaky and colleagues highlighted that truly inactive control conditions are limited by practical and ethical considerations [7].

Lower resource interventions targeting suicidal behaviour can be effective. For example, Caring Contacts is a low resource intervention involving sending supportive messages (e.g., postcards, phone calls, emails) to individuals post-SA [24]. While Caring Contacts has shown reduced suicidal behavior in adults, especially veterans [12]there is limited evidence for adolescents [25, 26]. Rengasamy and Sparks (2019) evaluated the impact of brief telephone follow-ups for adolescents following discharge from the Emergency Department (ED) or an inpatient unit; an intervention resembling the Caring Contacts intervention. Their results demonstrated that multiple calls (up to 6) were associated with a lower rate of suicidal behavior and more confidence in safety plans relative to a single call [27].

The performance of brief interventions in trials has been more variable than the more intensive treatments, possibly related to inclusion of adolescents presenting with relatively high acuity suicidality (e.g., recent SA). Given the high mortality and morbidity of adolescent suicidal behavior, early intervention when SI is less acute, may lead to improved responsiveness to brief treatment, reduced distress, and could potentially be lifesaving. The extant literature provides important guidance regarding components of successful interventions. Evidence to date suggests that interventions for adolescents with SI should incorporate safety planning (e.g., SAFETY program), skill building for individuals and caregivers (e.g., DBT, SAFETY), and improving the adolescent-caregiver relationship (e.g. ABFT). Similarly, Hughes and colleagues highlighted the importance of enhancing coping skills, targeting behaviour change, and fostering connections with supports (family and/or peers) [28]while also recommending a more multicomponent approach [13].

BRAVA is a manualized, brief group treatment that incorporates elements of established evidence-based interventions and the components highlighted by Hughes and colleagues [28]. BRAVA involves six weekly youth and caregiver groups focused on family connection to decrease suicidal behavior and increase family cohesion for youth with mild-to-moderate SI. Youth and caregivers attend separate groups, and each module is independent of the other to allow participants to join the group at any point in the 6-week sequence. The youth modules include activities encouraging reflection on adolescent-caregiver relationship, DBT skills (distress tolerance and mindfulness skills, validation), CBT skills (challenging negative thoughts), conflict resolution and crisis management. The caregiver modules have a consistent focus on validation alongside psychoeducation regarding adolescent development, communication skills, conflict resolution and crisis management. A BRAVA pre-post pilot study showed promising results, indicating clinically significant improvements in SI and other MH-related outcomes for adolescents and caregivers [29]. A subsequent virtual adaptation piloted during the COVID-19 pandemic demonstrated feasibility, acceptability, and high participant satisfaction [30]. BRAVA’s unique design intends to help minimize barriers to care. Its brevity, group format, and rolling entry allows prompt access. Manualized content facilitates delivery by clinicians with varied backgrounds and levels of experience. Virtual delivery reduces barriers related to geographic location and permits participation by families residing far from treatment facilities.

The primary objective of this study was to assess the efficacy of BRAVA for adolescents with mild-to-moderate SI and their caregivers in reducing adolescent SI compared to ETU. Secondary clinical outcomes were changes in youth depression and anxiety, youth and caregiver perceived stress, caregiver perception of general family functioning, adolescent-caregiver attachment, visits to the ED for psychiatric-related concerns, and treatment satisfaction.

## Method

### Trial design

Our RCT had an allocation ratio of 1:1 and parallel groups. The study was approved by the hospital institutional review board, conformed to CONSORT guidelines (Supplementary Material 1), and overseen by a Data Safety Monitoring Board consisting of members independent from the project. All adolescent and caregiver participants provided informed consent, and the study was registered with Clinicaltrials.gov (NCT04751968).

### Participants

Families were recruited from a pediatric hospital and community-based MH services in Ontario, Canada between April 2021 and April 2023. Eligible adolescents were screened by their provider at any point during their service journey (e.g., ED visit, community intake, primary care appointment, etc.) and, with consent, referred to the study for further eligibility screening. Adolescents were eligible for recruitment if they were 13- to 17.5-years-old, presented with mild-to-moderate SI (defined as a rating of 1 on the HEADS-ED [31]; and a score of 23 or above on the Suicidal Ideation Questionnaire Junior [SIQ-JR] [32]), interested in study participation, and had access to a suitable electronic device (Internet, camera, microphone). Exclusion criteria included SI with plan or gesture (rated as a 2 on HEADS-ED), comorbid disorders (psychosis, schizophrenia, severe developmental disabilities, major substance abuse, and severe eating disorders), externalizing disorder as a primary diagnosis or primary concern (e.g. Conduct Disorder), weekly psychotherapy, child protective services involvement, and/or expressed difficulty with reading and writing. Youth with comorbid and/or primary externalizing disorders were excluded from the study as previous research demonstrates this population may not benefit from group therapy as it requires adequate cognitive capacity and/or self-regulation to be able to benefit from group participation [33].

**Fig. 1:**
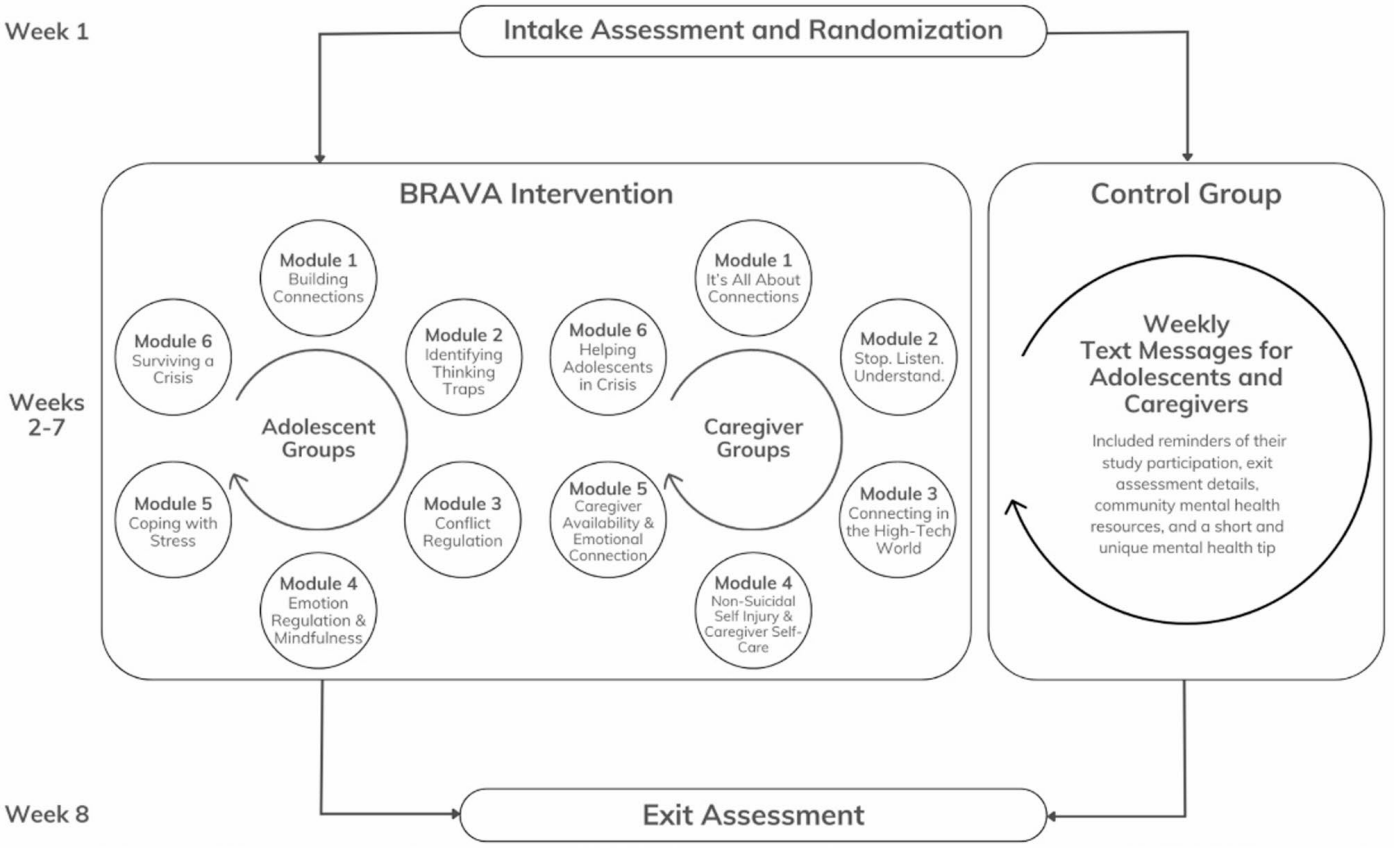
Study process and intervention and control group characteristics

Figure 1 outlines the study timeline for participants. The study intakes and exits were conducted by a graduate-level RA trained in suicide risk assessment. These assessments included eligibility screening, safety planning, and resource recommendations for all participants. A Psychologist or Psychiatrist was on-call to assist with any safety concerns for all assessments.

### Randomization

Eligible participants were randomized in REDCap [34]. An independent statistician produced a computer-generated allocation table using randomly alternating block sizes of 4 and 6. Allocation was concealed during the eligibility screening process and study staff were made aware of the permanent, group allocation post-randomization. Blinding to treatment allocation was not possible for study staff or participants as the intake occurred prior to randomization.

### Interventions

#### BRAVA group intervention

BRAVA families started the intervention within 6.6 days (SD = 5.2) of completing their intake on average. The six-week, 90-minute youth and caregiver groups were held on separate days. Table 1 highlights the goals and content for youth and caregiver groups. The youth group incorporated CBT and DBT skills, mindfulness and fostering family connection, and were co-facilitated by a Psychologist and a Child and Youth Counsellor. The caregiver group included psychoeducation on adolescent development, conflict management, importance of validation and crisis de-escalation, and were co-facilitated by one to two Child and Adolescent Psychiatrist(s) and a Research Associate.

**Table 1:** BRAVA modules

Youth		Caregivers	
Module	Skills and Topics	Module	Skills and Topics
1: Building Connections	- Importance of connections - Improving connection with parents - Peer vs. family social support	1: It’s All About Connections	- Importance of connections - Psychological and biological adolescent development - Barriers to connection - Validation
2: Identify Cognitive Bias	- The connection between thoughts, feelings, and behaviors - Challenging distorted/maladaptive thoughts	2: Stop. Listen. Understand.	- Effective communication - Listening - Parenting styles
3: Conflict Resolution: In Person and Online	- Conflict resolution - Bullying - Social media and internet safety	3: Connecting in the High-Tech World	- Technology - Digital safety - Social media and wellbeing - Family media plan
4: Emotion Regulation and Coping	- Mindfulness - Radical acceptance	4: Caregiver Self-Care and Stress, and Non-Suicidal Self-Injury	- Non-suicidal self-injury - Caregiver stress management - Self-care
5: Distress Tolerance and Mindful Acceptance	- Healthy coping - Understanding impact of stress - Developing a sleep routine	5: Caregiver Availability and Emotional Connection	- Problem-solving - Understanding executive functioning and lagging skills
6: Surviving a Crisis	- Crisis management - ACCEPTS - Coping thoughts - Safety planning - Suicidal thoughts	6: Helping Adolescents in Crisis	- Managing a crisis - Suicidal behaviour - Suicide check-in - Protective factors - How to seek help

Adolescent and caregiver modules ran on separate days

The modules were designed to be independent, allowing families to enter and exit at any point during the 6-module cycle. Both groups had a RA assigned for administrative and technology activities (tracking group attendance, PowerPoint slides). Full manual including the intervention modules are available upon request by contacting the corresponding author. Group attendance was reported as the number of sessions attended out of six for youth and caregivers.

#### Treatment fidelity

To ensure that group leaders adhered to the treatment manual, fidelity checklists were developed for the intervention and all sessions were audio-recorded. Recordings for all groups in the first three rounds of BRAVA (36 groups) had fidelity coding completed by a trained research assistant not affiliated with the project to ensure reliability which demonstrated a 98.6% correspondence to the treatment manual. For the remainder of the RCT, 25% (*n* = 38/152 groups) were randomly selected for fidelity coding and reliability was maintained with 97.8% (Range 75–100%) correspondence with the treatment manual.

#### Enhanced treatment-as-usual control group

The ETU control group included a weekly text message intervention and the opportunity to participate in the BRAVA intervention once the family had completed their exit assessment. The messages were modeled after the Caring Contacts intervention [35] including reminders of their exit appointment, community resources, and a different and short MH tip (Table 2). Participants were able to reply to the text messages. However, participants were made aware that messages were not monitored 24/7 and was not a crisis line, and was limited to strictly communications around study-related activities (e.g., weekly messages, scheduling and reminders of assessments, surveys, etc.).

**Table 2:** Summary of short mental health tips included in weekly ETU text messages

Week	Mental Health Tip
1	“Some people find that taking a break from social media helps them feel better.”
2	“Living in the moment can help your mental health.”
3	“Telling yourself something like “I can get through this” can help you deal with stress.”
4	“Exercising or going outside can help you feel better.”
5	“Doing something nice for someone else can help you feel more positive.”
6	“Getting a good night’s sleep can help you manage your feelings.”

#### Assessments

Before the intake, the adolescent and the identified primary caregiver provided informed consent and completed secondary outcome measures via REDCap. The 60-minute intake assessment included an overview of the study, completion of the HEADS-ED [31, 36] SIQ-JR [32] a suicide risk assessment, and safety planning. Most of the assessment was completed with the youth alone, and the caregiver was instructed to remain in the home should safety concerns arise. If eligible, the family was randomized to the BRAVA group (*n* = 50) or the ETU Control group (*n* = 49).

**Fig. 2:**
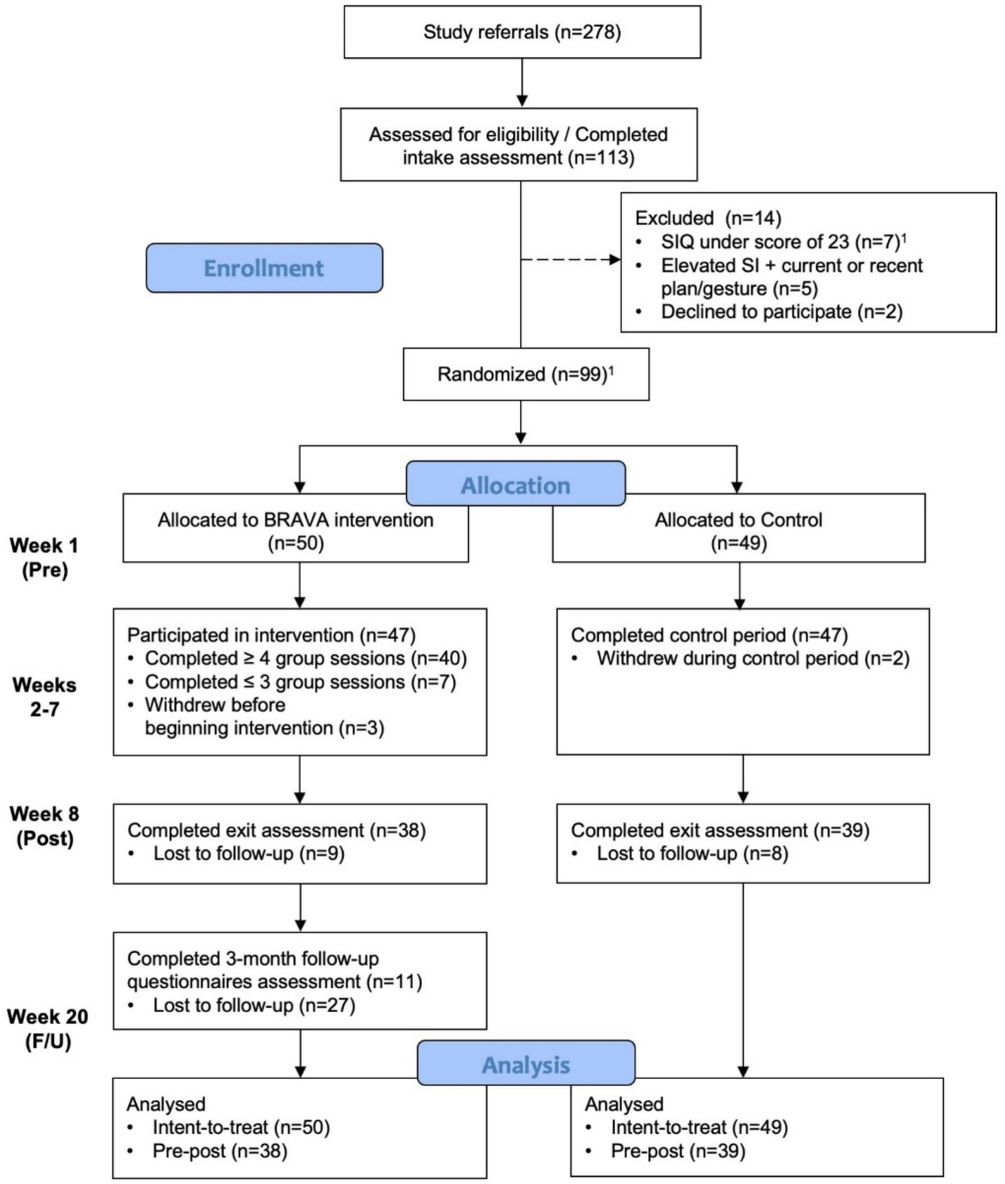
CONSORT flowchart of participants comparing BRAVA with ETU control group [1]. The inclusion criteria of a score of 23 or above on the SIQ-JR was added 6-weeks into the trial after 2 participants who met original inclusion criteria had exceptionally low scores on the SIQ-JR. Data from these participants remained in the analysis

Following their BRAVA or ETU Control group participation, families completed an exit assessment which included the same procedures as the intake. Follow-up questionnaires were emailed to BRAVA families 12-weeks after their exit assessment via REDCap. ETU Control group participants did not complete 12-week follow-up questionnaires as they were invited to participate in the BRAVA intervention following their exit assessment. See Fig. 2 for study flow. Adolescent study participants received a $25 gift card once participation was complete.

### Measures

#### Adolescent measures

HEADS-ED [31, 36]: The HEADS-ED was used to assess for study inclusion criteria for suicidality and to describe the level of functioning/need in study participants. It is a validated, seven-item MH screening tool for children and youth that assesses the level of need and functioning across seven domains. The items are scored on a scale of 0 (*no action needed*), 1 (*needs action but not immediate/moderate functional impairment*), or 2 (*needs immediate action/severe functional impairment*). Items can stand on their own or be tallied into a total score.

SIQ-JR [32]: The SIQ-JR is a 15-item version of the SIQ for adolescents aged 15–18. The SIQ-JR assesses SI within the last month on a scale of 0 (*I never had this thought) to* 6 *(Almost every day*) with a 3-week test-retest reliability of 0.89^39^. A score of 23 or higher was required for study inclusion criteria as, based on normative data, it is one standard deviation below the mean. A score of 31 or greater is above the clinical cut-off.

MH Service History: This one-item scale was developed by the research team and RAs rated the adolescent’s MH Service History on a scale of 1 (*No history of MH services/BRAVA is their first MH treatment experience)* to 4 (*History of intense MH service use*).

#### Caregiver measures

Adolescent Anxiety and Avoidant Attachment Inventory– Long Form Parent Report of Youth (AAAAI) [38]: The AAAAI is a reliable and valid [39] 36-item questionnaire used to evaluate adolescent-parent attachment within the last month. Items are rated on a 7-point scale 1 (*Strongly Disagree)* to 7 (*Strongly Agree*). Items are scored into Attachment Anxiety and Attachment Avoidance subscale with higher scores indicating higher attachment anxiety or avoidance.

Family Assessment Device (FAD) [40]: The FAD is a psychometrically sound 60-item self-report measure used to assess family functioning based on a 4-point scale from 1 (*Strongly Agree*) to 4 (*Strongly Disagree*). An average rating is determined for each subscale. Only the 12 item General Functioning subscale was used for this study.

#### Adolescent and caregiver measures

Demographic data was collected from the youth (age, gender, ethnicity, and current use of psychotropic medications) and caregivers (age, relation to child, annual household income, and MH history). The ethnicity response options were aligned with the Government of Canada’s 2016 Census Profile [41].

Revised Child Anxiety and Depression Scale (RCADS) [42]: The RCADS is a psychometrically sound 47-item questionnaire that screens for adolescent depression and anxiety and can be completed by adolescents and/or caregivers. Items are scored from 0 (*Never)* to 3 (*Always*). Only the Total Depression and Anxiety score were used for this study.

Perceived Stress Scale (PSS) [44]: The PSS is a reliable 10-item self-report questionnaire assessing the level of stress one perceives for their life with items rated on a 5-point Likert scale ranging from 0 (*Never) to* 4 (*Very Often*) where a higher score indicates a higher level of perceived stress and is categorized as low (0–13), moderate (14–26), or high (27–40) perceived stress.

Perceived Group Cohesion (PGC) [46]: The PGC is a reliable 6-item scale assessing a participant’s sense of morale and belonging in a small group. Items are rated on a 7-point Likert scale ranging from 1 (*Strongly Disagree)* to 7 (*Strongly Agree*) with higher scores indicating a higher perceived cohesion within a group.

BRAVA Satisfaction: Participants were asked to provide qualitative feedback during their exit assessment about their overall experience with BRAVA. A reflexive thematic analysis was conducted to organize and describe the data in detail by determining recurring patterns and themes [47]. Without any pre-defined categories, we used an inductive exploration approach to determine themes by following the six recommended steps for reflexive thematic analysis, including (1) familiarize yourself with the data; (2) conduct initial coding; (3) generate initial themes; (4) review the developed themes; (5) refine and define themes, then (6) summarize for reporting [48]. The data was coded by two independent coders with a third coder resolving conflicts.

### Statistical analysis

The distribution of population characteristics and outcomes was summarised using proportions or means and standard deviations. Intention to treat (ITT) analysis was performed for youth and caregiver cohorts. We used Multiple Imputation by Chained Equations (MICE) to impute the following outcomes with missing data at their follow-up times: Adolescent SIQ-JR, Adolescent PSS, Adolescent RCADS Total and Major Depression scores, Adolescent FAD General Functioning, Caregiver RCADS Total Score, Caregiver AAAAI Avoidance and Anxious/Insecure Scores, Caregiver PSS, Caregiver FAD Total and General Functioning Scores. All baseline characteristics including baseline values of the outcomes were initially considered for the imputation model. Once the fit of the imputation model was considered acceptable, a linear regression analysis was performed. For each of the outcomes, we first checked for homogeneity of regression slope by testing an interaction term between treatment allocation and the score at baseline. If the homogeneity of slopes was confirmed, we fit a linear regression model without interaction term to obtain the effect of treatment allocation on the post-intervention score (time 2) adjusted for the score at baseline (time 1). The results are presented in terms of post-intervention means adjusted for scores at baseline. For the BRAVA group, a linear mixed effect model was also fit to estimate the mean change in SIQ-JR score over time (pre, post, and 3-month follow-up). Homogeneity of regression slopes was assessed before fitting a final model. Estimated marginal means in the two groups were obtained using ANCOVA analysis. Homogeneity of variances was checked using Levene’s test. Standardized residuals greater than 3 in absolute value were used to check for possible outliers. In addition, we conducted a sensitivity analysis for the change in SI measured by the SIQ-JR for youth who completed both pre- and post-treatment assessments.

### Sample size

Based on a priori power analysis, a sample size of 47 to 58 was determined necessary to achieve a comparable effect size to previous research on SI interventions (i.e., f = 0.485)^13^, with 90 to 95% power at α =.05^51^, However, given that between 28 and 75% of youth MH treatments are terminated prematurely due to attrition [50] the targeted recruitment sample was inflated to 98 youth to account for equal group allocation and anticipated dropouts.

## Results

Of 278 families referred to the study, 113 (40.6%) consented and participated in an intake assessment. Fourteen were identified as ineligible (primarily due to SIQ-JR not falling in target range) at intake, and 99 were randomized (*n*_BRAVA_=50; *n*_Control_=49). Approximately three quarters of the sample was retained for their exit assessment (BRAVA = 74%, Control = 79.6%). Almost one third of BRAVA participants who completed the exit assessment also completed the 3-month follow-up questionnaires (29.7%). See Fig. 2 for full participant flow. Group session attendance was high for both youth and caregivers [youth Median (Mdn) = 6, caregiver Mdn = 5]. Total PGC was moderately high for both youth (M = 4.9, SD = 1.6) and caregivers (M = 5.7, SD = 0.3). No significant differences were found at baseline when comparing age (*p* = 0.212), gender (*p* = 0.839) and history of MH service access (*p* = 0.345) for BRAVA participants who completed a minimum of 4 groups versus those who did not.

### Baseline data

At baseline, youths averaged 14.6 years, identified mostly as female (64%), English as their primary language (70%). Almost half (44%) self-identified as European heritage and 28% reported no history of MH services. Both groups presented with comparable scores for all clinical outcome measures, and most were in the clinical range. See Tables 3 and 4 for a description of the study cohort.

**Table 3:** Baseline description of the cohort– youth (*N* = 99)

Youth Characteristics	BRAVA, *n* = 50	Control, *n* = 49	Total, *N* = 99	
	M (SD)	M (SD)	M (SD)	
Age (years)	14.6 (1.4)	14.6 (1.1)	14.6 (1.2)	
	**n (%)**	**n (%)**	**n (%)**	
Gender				
Male	10 (20.0)	7 (14.3)	17 (17.2)	
Female	32 (64.0)	32 (65.3)	64 (64.6)	
Other	8 (16.0)	10 (20.4)	18 (18.2)	
Language spoken at home				
English	35 (70.0)	39 (79.6)	74 (74.5)	
French	4 (8.0)	2 (4.1)	6 (6.1)	
English and French	7 (14.0)	5 (10.2)	12 (12.1)	
English and Other	2 (4.0)	3 (6.1)	5 (5.1)	
Primarily Other	2 (4.0)	0 (0.0)	2 (2.0)	
Ethnicity/Racial heritage				
European	22 (44.0)	20 (40.8)	42 (42.2)	
Asian	3 (6.0)	4 (8.2)	7 (7.1)	
Latin, Central South American	5 (10.0)	0 (0.0)	5 (5.1)	
North American Aboriginal	2 (4.0)	4 (8.2)	6 (6.1)	
African	3 (6.0)	1 (2.0)	4 (4.0)	
Caribbean	1 (2.0)	2 (4.1)	3 (3.0)	
Multiple race(s) reported	6 (12.0)	3 (6.1)	9 (9.1)	
Other	8 (16.0)	15 (30.6)	23 (23.2)	
History of mental health service use				
No	14 (28.0)	16 (32.7)	30 (30.3)	
Yes	36 (72.0)	33 (67.3)	69 (69.7)	
Currently taking psychotropic medication				
No	15 (30.0)	13 (26.5)	28 (28.3)	
Yes	35 (70.0)	36 (73.5)	71 (71.7)	
HEADS-ED Domains Home				
Supportive (0)	31 (62.0)	26 (53.1)	57 (57.6)	
Conflicts (1)	18 (36.0)	23 (46.9)	41 (41.4)	
Chaotic/Dysfunctional (2)	1 (2.0)	0 (0.0)	1 (1.0)	
Education				
On track (0)	19 (38.0)	17 (34.7)	36 (36.4)	
Grades dropping/absenteeism (1)	28 (56.0)	25 (51.0)	53 (53.5)	
Failing/not attending school (2)	3 (6.0)	7 (14.3)	10 (10.1)	
Activities				
No change (0)	22 (44.0)	13 (26.5)	35 (35.4)	
Reduced/peer conflicts (1)	24 (48.0)	32 (65.3)	56 (56.6)	
Fully withdrawn/significant peer conflicts (2)	4 (8.0)	4 (8.2)	8 (8.1)	
Drugs and alcohol				
None or Infrequent (0)	41 (82.0)	39 (79.6)	80 (80.8)	
Occasional (1)	9 (18.0)	10 (20.4)	19 (19.2)	
Frequent/daily (2)	0 (0.0)	0 (0.0)	0 (0.0)	
Suicidality				
No thoughts (0)	0 (0.0)	0 (0.0)	0 (0.0)	
Ideation (1)	50 (100.0)	49 (100.0)	99 (100.0)	
Plan or gesture (2)	0 (0.0)	0 (0.0)	0 (0.0)	
Emotions, behaviors, thought disturbance				
Mildly anxious/sad/acting out (0)	4 (8.0)	2 (4.1)	6 (6.1)	
Moderately anxious/sad/acting out (1)	38 (76.0)	41 (83.7)	79 (79.8)	
Significantly distressed/unable to function/out of control/bizarre thoughts (2)	8 (16.0)	6 (12.2)	14 (14.1)	
Discharge resources				
Ongoing/well connection (0)	2 (4.0)	5 (10.2)	7 (7.1)	
Some/not meeting needs (1)	35 (70.0)	30 (61.2)	65 (65.7)	
None/on waitlist/non-compliant (2)	13 (26.0)	14 (28.6)	27 (27.3)	
HEADS-ED Total Score	5.2 (1.3)	5.6 (1.4)	5.4 (1.36)	
SIQ-JR	50.0 (16.1)	56.1 (16.1)	53.0 (16.6)	
RCADS Total Score	79.6 (13.7)	83.0 (13.7)	81.3 (13.7)	
PSS	29.6 (4.9)	30.6 (3.9)	30.1 (4.5)	

*M* mean, *SD* standard deviation, *SIQ-JR* suicide ideation questionnaire-junior, *RCADS* revised child anxiety and depression scale, *PSS* perceived stress scale

**Table 4:** Description of the cohort– caregiver (*N* = 99)

Caregivers Characteristics	BRAVA, *n* = 50	Control, *n* = 49	Total, *N* = 99
	M (SD)	M (SD)	M (SD)
Age	46.3 (6.6)	46.9 (7.3)	46.6 (6.9)
	**n (%)**	**n (%)**	**n (%)**
Sex			
Male	11 (22.0)	7 (14.3)	17 (17.2)
Female	37 (74.0)	40 (81.6)	79 (79.8)
Other	0 (0.0)	1 (2.0)	1 (1.0)
Missing	2 (2.0)	1 (2.0)	3 (3.0)
Relationship to the child in treatment			
Father (birth, step-, adoptive, foster, god-)	11 (22.0)	7 (14.3)	18 (18.2)
Mother (birth, step-, adoptive, foster)	38 (76.0)	41 (83.7)	79 (79.8)
Missing	1 (2.0)	1 (2.0)	2 (2.0)
RCADS Total (t-score)	81.9 (16.4)	80.8 (18.1)	81.4 (17.2)
PSS	21.2 (6.2)	19.81 (5.3)	20.53 (5.8)
FAD	1.91 (0.5)	1.94 (0.4)	1.93 (0.5)
AAAAI Anxiety	3.08 (1.1)	2.80 (1.1)	2.94 (1.1)
AAAAI Avoidance	2.90 (1.3)	3.27 (1.4)	3.09 (1.4)

*M* mean, *SD* standard deviation, *RCADS* revised child anxiety and depression scale, *PSS* perceived stress scale, *FAD* family assessment device, *AAAAI* adolescent anxiety and avoidant attachment inventory.

### Primary outcome

**Fig. 3:**
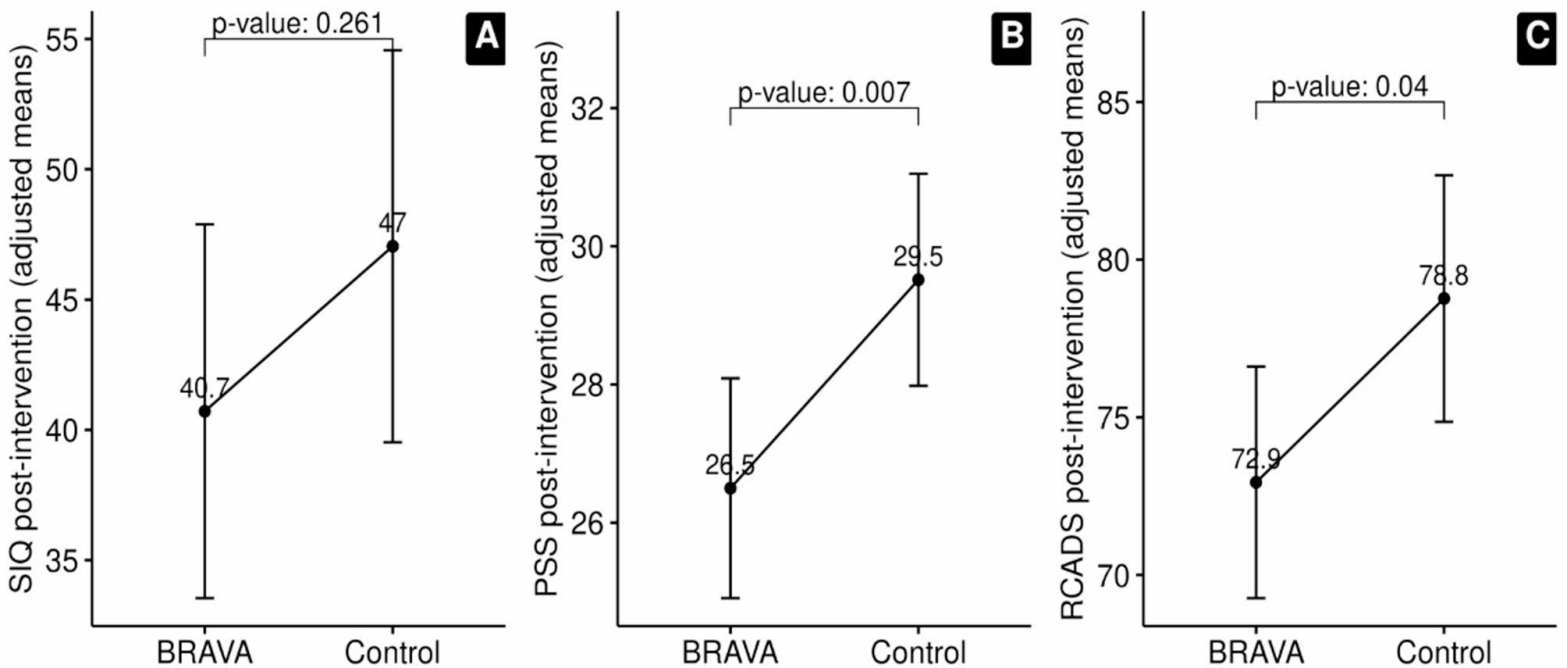
Youth Outcomes. **A** SIQ-JR post-intervention estimated marginal means adjusted for baseline score. **B** PSS post-intervention estimated marginal means adjusted for baseline score. **C** RCADS post-intervention estimated marginal means adjusted for baseline score

SI was elevated across groups at baseline, with 84% of BRAVA group and 92% ETU control scoring above the SIQ-JR’s clinical cutoff. In ITT analyses, there was no statistically significant difference in youth SIQ-JR post-intervention scores between the BRAVA (estimated marginal mean [EMM] = 40.7) and ETU Control groups (EMM = 47.0; t(96)=-1.146, *p* = 0.261, η_g_^2^ = 0.034); Fig. 3A). Sensitivity analysis evaluating only those youth with pre- and post-SIQ-JR data (i.e., no multiple imputations for missing data) did show a statistically significant difference in SIQ-JR post-intervention scores between the groups [BRAVA EMM = 41.9, ETU control EMM = 49.6; F(1,73) = 5.03, *p* = 0.028, η_g_^2^ = 0.06].

**Fig. 4:**
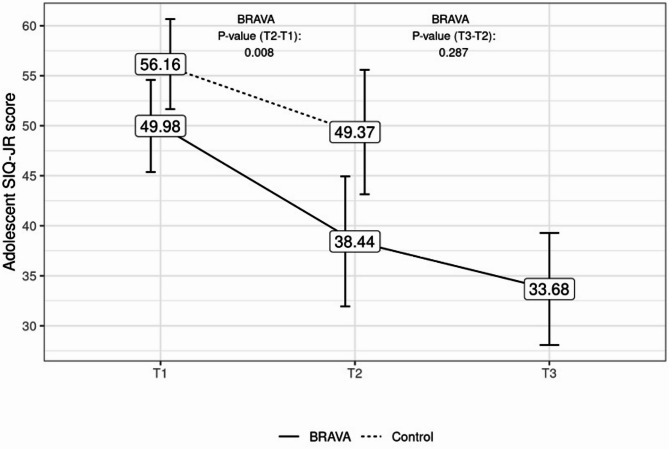
Youth SIQ-JR Scores for BRAVA and Control participants. Mean BRAVA and Control group SIQ-JR scores at Time 1 (T1; pre-treatment), Time 2 (T2; post-treatment), and BRAVA Time 3 (T3; 3-month follow-up)

The percentage of youth in the clinical range on the SIQ-JR decreased by 25% between pre- (84%) and post-treatment (59.5%) for the BRAVA group compared to 10% for the ETU control group (pre = 91.8%, post = 82.1%). Three-month follow-up data was available for 11 (29.8%) BRAVA participants (Fig. 4). The mean SIQ-JR scores for the BRAVA group significantly decreased between pre- and post-intervention from 49.98 to 38.44 (*p* = 0.008). Average post-treatment decreases on the SIQ-JR were maintained at 3-month follow-up (33.68; *p* = 0.287) with no significant change in scores observed. There were no significant differences in baseline characteristics between those who completed their follow-up questionnaires and those who did not. No follow-up ETU group data was collected per the study design.

### Secondary youth outcomes

After adjustment for baseline scores, there was a statistically significant difference in PSS post-intervention scores between the treatment and control groups [BRAVA EMM = 26.5 (medium level of perceived stress), ETU control EMM = 29.5 (high level of perceived stress); t(96)=-2.770, *p* = 0.007, η_g_^2^ = 0.082] and RCADS Total Depression and Anxiety t-score [BRAVA EMM = 72.9, ETU control EMM = 78.8; t(96)= -2.100, *p* = 0.04, η_g_^2^ = 0.058]. See Fig. 3, panels B and C.

### Caregiver outcomes

There were no significant differences for any of the caregiver post-intervention outcomes adjusted for baseline scores. Mean RCADS Total Depression and Anxiety post-intervention t-scores remained in the clinical range for both groups [BRAVA EMM = 75.9, ETU control EMM = 74.9; t(96) = 0.309, *p* = 0.758, η_g_^2^ = 0.004]. Other outcomes measures included family functioning and attachment as measured by the FAD [BRAVA EMM = 1.86, ETU control = 1.93, t(96)=-0.708, *p* = 0.481, η_g_^2^ = 0.012], the AAAAI Avoidance [BRAVA EMM = 3.05, ETU control = 3.15, t(96)=-0.445, *p* = 0.658, η_g_^2^ = 0.008], AAAAI Anxious/insecure scales [BRAVA EMM = 3.04, ETU control = 3.14, t(96)=-0.496, *p* = 0.621, η_g_^2^ = 0.005], and Perceived Stress [BRAVA EMM = 19.5, ETU control = 21.7, t(96)=-1.791, *p* = 0.077, η_g_^2^ = 0.04].

### ED visits

Throughout study participation, fourteen youth presented to the ED for psychiatric-related concerns (n_BRAVA_=6, n_Control_=8, t(97) = 0.61, *p* = 0.54). The number of these ED visits was significantly higher in the ETU control group than the BRAVA group over the pre-post study period (n_BRAVA_=7, n_Control_=17, t(97) = 2.45, *p* = 0.02) and during the 3-month follow-up (n_BRAVA_=6, n_Control_=14, t(97) = 2.08, *p* = 0.04). All visits were classified as expected adverse events.

### Treatment satisfaction

Having a positive experience with BRAVA was the most recurrent theme among participants at the exit assessment. Most adolescents and caregivers reported BRAVA to be beneficial and helpful to them, and that they learned new skills from the group (e.g., thinking traps for adolescents, validation for caregivers). One adolescent enjoyed “getting a lot of information and resources about what to do and how to cope with suicidal ideations”, while a caregiver reflected on the importance of listening to and not problem solving for their youth noting “it was such a great eye opener to teach us not to problem solve and view things in a different perspective”. Some participants discussed the sense of having peer support in BRAVA as they were able to learn from other group participants and understand they were not alone in their experience. One youth expressed liking BRAVA because “everyone had similar thoughts, and no one would think that one person had weird thoughts,… or no one would feel left out”, while a caregiver found it helpful to “hear from other parents [and] hear things they are trying”. Some participants did not find all the intervention strategies useful but were still able to describe aspects of the intervention they enjoyed (e.g., discussing with peers in similar situations).

## Discussion

This study demonstrated that this brief group intervention may be beneficial for adolescents with mild-to-moderate SI. Although the ITT analysis of the primary outcome, SI, did not reach statistical significance, the between-group difference in post-intervention SI adjusted for baseline SI favored the BRAVA group. For two secondary outcomes, youth perceived stress and a combined measure of anxiety/depression, between-group comparisons also favored the BRAVA group, and reached statistical significance. The caregiver’s perceived stress, family functioning, and attachment did not demonstrate a significant treatment effect. Additional sensitivity analysis, excluding multiple imputations for missing data, revealed a significantly greater reduction in SI among BRAVA participants following treatment. However, there may be a risk of bias due to differences between dropouts and those who remained. Notably, 25% of BRAVA completers, compared to 10% of ETU participants, no longer had clinically elevated scores on the SIQ-JR at their exit assessment.

Both BRAVA participants and members of the ETU group experienced significant improvements in depression/anxiety and perceived stress as evidenced by improvements in their scores on related outcome measures. Of note, the ETU group was an active control design adapted from Caring Contacts, a low-resource intervention which has demonstrated reductions in SI and behaviour in previous studies [24]. Within-group analyses revealed that, BRAVA participants’ improvements in SI were maintained at 3-month follow-up. When compared to ETU, BRAVA participants also had significantly fewer ED presentations during the study period. BRAVA youth and caregiver participants reported high group cohesion, indicating a sense of belonging and morale within the intervention group. These results, in conjunction with the finding that BRAVA participants sustained treatment gains over 3-months and had significantly fewer ED visits during the study period (compared to ETU), are encouraging findings regarding BRAVA’s efficacy. This study extends previous findings, including a pre-post study demonstrating improvements in SI, mood, anxiety, and caregiver stress with BRAVA [29] and a pilot study on the virtual adaptation of BRAVA, which found it to be feasible, acceptable, and associated with moderately high participant satisfaction [30].

Most interventions that have shown clinical effectiveness in reducing SI (e.g., DBT, ABFT) are lengthy, resource-intensive treatments. These treatments are tailored for adolescents with acute suicidal symptoms. This study targeted adolescents with mild-to-moderate SI but baseline SIQ-JR scores suggested a similar level of suicidality compared to previous ABFT and DBT trials, with most participants falling in the clinical range. Recruitment sources (primarily outpatient settings) and the percentage of adolescents taking psychotropic medication (72% of participants) also suggest a comparable level of acuity in our sample [8, 51]. Regardless, participants in the BRAVA intervention demonstrated clinically meaningful improvements, as indicated by fewer ED visits and lower scores post-treatment on their self-reported outcome measures.

Given the volume of adolescents seeking intervention for suicidal behavior and the need to minimize wait times [21] there is a need to develop brief treatments with minimal barriers. BRAVA’s six-week group format may fill a service gap of evidence-based interventions for youth with SI and their caregivers. Studies on similarly brief interventions are limited with emerging evidence regarding clinical effectiveness. In a clinical noninferiority trial, a brief IPT-based suicide crisis intervention for adolescents yielded reductions in SI and behavior similar to a lengthier treatment but neither treatment outperformed the control group [23]. The authors mused that the involvement of RAs and the hope of future intervention may have benefited participants. The presence of active control groups in suicide intervention studies combined with low power pose considerable challenges in obtaining significant treatment outcomes in RCTs [7]. The control group used for this study is no exception as it was similar to a Caring Contacts intervention, involving weekly text messages to participants and the opportunity to join the treatment group following study participation. Caring Contacts interventions have shown promise in adult populations but research with adolescents remains limited [24]. Beyond symptoms of SI, perceived stress has not been consistently measured in ABFT and DBT studies. The improvements in adolescent perceived stress observed in this study are consistent with studies of Intensive Contextual Treatment with self-harming and suicidal adolescents and their families [52].

In this study, high satisfaction and group cohesion outcomes suggest that the caregiver sample derived benefits from BRAVA in the absence of a significant treatment effect for caregiver attachment, family functioning, and perceived stress outcomes. In other SI-related studies, caregiver attachment is not commonly measured.. Nevertheless, our attachment scores at intake and exit are comparable to Moretti et al. (2015), who evaluated the effectiveness of an attachment-based intervention for caregivers of at-risk adolescents [53]. This raises the question of whether measuring attachment is feasible within brief interventions as it may require more than six weeks to demonstrate change in family-based studies for adolescents with SI. Moreover, a meta-analysis of group cohesion in adult group therapy interventions previously found a mildly positive correlation between cohesion and outcomes in groups containing 5–9 members [54]. While our caregiver outcomes were nonsignificant between groups, our group cohesion findings may potentially indicate benefits in other unexplored domains, such as resilience or parenting confidence, and should be explored in future research. Participant satisfaction with the intervention mirrored previously published BRAVA studies, with most participants expressing positive feedback and highlighting peer support and skill acquisition for youth to support themselves and for caregivers to support their youth [29, 30]. Additionally, moderately high levels of group cohesion have been observed among youth and caregivers in DBT studies [55, 56].

In addition to reducing SI symptoms, BRAVA aims to minimize wait times for adolescents experiencing SI. Its rolling entry feature allows families to enter at any point in the 6-week cycle and within a week after their intake, distinguishing it from other evidence-based interventions for adolescent SI and ensuring prompt treatment. Moreover, BRAVA is a manualized program which can be easily implemented in hospital or community settings. It incorporates elements effective in reducing SI, including safety planning, core coping skills, a focus on fostering connections, and parental involvement. Participants expressed that hearing from others with similar challenges reduced feelings of isolation, and group discussions supported emotional management. While DBT also includes these elements, it operates within a more intensive and costly framework.

With consideration to study limitations, despite a diverse sample in terms of ethnicity and gender, youth participants were predominantly white females, with caregivers being primarily mothers, potentially limiting the generalizability of findings. Encouragingly, we found no differences between groups at baseline, which indicates low risk for selection bias. Our study shares similarities with previous SI-related research, particularly in terms of participant demographics (e.g., predominantly female and white, use of psychotropic medication) [8, 11–14, 23, 27]. Our study population consisted of youth with mild-to-moderate SI and excluded those with a primary externalizing disorder and/or comorbid disorder, which may impact the generalizability of our findings in populations with more acute MH concerns also experiencing SI. 

One quarter of BRAVA participants and one fifth of ETU controls were lost to post-treatment follow-up, necessitating imputing missing data to mitigate attrition bias. Nonetheless, participant follow-up rates were consistent with other adolescent SI-related studies, which have high variability ranging from 0 to 40%^8,7^. The lack of 3-month follow-up measures for the ETU group based on the study design and the elevated 3-month BRAVA participant loss to follow-up makes it difficult to conclude any possible treatment maintenance or improvement effects. Furthermore, not all participants opted to provide qualitative feedback on their satisfaction with the intervention which may indicate a potential courtesy bias in our findings, whereby participants may have not reported negative feedback to be polite or avoid offending the research team. However, our findings are consistent with our previous work on BRAVA which further increases our confidence in these results [29, 30]. 

Given the ethical and retention implications of conducting research with a highly vulnerable population in need of MH treatment, we purposefully chose not to mask the participants’ allocation for assessors at exit to ensure optimal safety assessments could be completed with the youth or collect 3-month follow-up data on control group participants to provide immediate BRAVA intervention following their exit from the control group. Group facilitators were highly experienced clinicians, raising uncertainty about treatment effects with more novice clinicians. However, the co-facilitator model used, and the manualized treatment may mitigate this limitation, while also aiding in training junior clinical staff and potentially reducing costs. Finally, SI significantly improved for BRAVA participants, but SIQ-JR scores remained in the clinical range for 59.5%. BRAVA may work best within stepped model of care, in which some adolescents proceed to more individualized treatment following BRAVA.

Future research should focus on the longer-term outcomes of treatment and the added value of various BRAVA features, such as rolling entry and separate youth and caregiver groups. For example, exploring the potential order effects of joining BRAVA at different points during the 6-week cycle may be beneficial. The feasibility of implementing the group in different populations and settings could also be explored. While BRAVA can be implemented in its entirety, some settings may benefit from implementation of stand-alone youth or caregiver modules. Recently, the youth modules have been implemented in a 30-day live-in treatment program for youth with complex mental health issues and the caregiver modules have been implemented in a psychiatric aftercare program. The efficacy of these modifications should be explored in future studies.

BRAVA may be a promising intervention for adolescents with SI. This brief group intervention was associated with significantly greater improvements in anxiety/depression and adolescent perceived stress compared to ETU for this study. BRAVA was associated with significantly fewer ED visits and yielded a higher percentage of participants who improved from clinical to non-clinical range on the SIQ-JR compared to the control group. Although BRAVA did not result in significant caregiver-reported improvements, group cohesion and treatment satisfaction were high for youth and caregivers. BRAVA’s brief and manualized content provides the opportunity for implementation by varied clinicians in a variety of settings.

## Electronic supplementary material

Below is the link to the electronic supplementary material.

Supplementary Material 1

## Data Availability

The data that support the findings of this study are not openly available due to reasons of sensitivity and are available from the corresponding author upon reasonable request.

## Abbreviations

AAAAI: Adolescent anxiety and avoidant attachment inventory– long form parent report of youth
ABFT: Attachment-based family therapy
CBT: Cognitive-behavioral therapy
DBT: Dialectical behavior therapy
ED: Emergency department
EMM: Estimated marginal means
ETU: Enhanced treatment-as-usual
FAD: Family assessment device
IPT-A-SCI: Interpersonal psychotherapy for adolescents
ITT: Intent to treat
M: Mean
Med: Median
MH: Mental health
MICE: Multiple imputation by chained equations
PGC: Perceived group cohesion
PSS: Perceived stress scale
RA: Research assistant
RCADS: Revised child anxiety and depression scale
RCT: Randomized controlled trial
SA: Suicide attempt
SD: Standard deviation
SE: Standard error
SI: Suicidal ideation
SIQ-JR: Suicidal ideation questionnaire– junior
